# Shoulder Synovial Chondromatosis Managed Arthroscopically in a Young Adult: A Case Report

**DOI:** 10.7759/cureus.100445

**Published:** 2025-12-30

**Authors:** Sumit Banerjee, Bharat Kumar Soni, Vimal Prakash, Furqan Meer, Abhay Elhence

**Affiliations:** 1 Orthopaedics, All India Institute of Medical Sciences, Jodhpur, Jodhpur, IND

**Keywords:** arthroscopy and arthroplasty, chondromatosis, chronic joint pain, shoulder joint pain, shoulder scope

## Abstract

Synovial chondromatosis is a rare benign joint disorder that can cause pain, stiffness, and restricted motion. Shoulder involvement is uncommon and may mimic other intra-articular conditions. A young adult male patient presented with chronic right shoulder pain and limited movement without prior trauma. Imaging revealed multiple intra-articular calcified bodies consistent with synovial chondromatosis. The patient underwent arthroscopic removal of loose bodies with partial synovectomy, during which around 20 fragments were extracted. Histopathology confirmed benign cartilaginous nodules without atypia. Postoperative recovery was uneventful, with early physiotherapy initiated. At three months, the patient achieved a full, pain-free range of motion and a significant improvement in functional scores. Follow-up imaging confirmed complete removal and no recurrence. This case highlights the role of arthroscopic excision and synovectomy as a safe, minimally invasive, and effective treatment for shoulder synovial chondromatosis, offering rapid recovery and excellent early outcomes.

## Introduction

Synovial chondromatosis is a rare, benign proliferative disorder of the synovium characterized by the development of multiple intra-articular cartilaginous nodules, most frequently involving large joints such as the knee and hip [1]. It typically presents with pain, swelling, and progressive limitation of joint motion. The underlying pathophysiology is attributed to synovial membrane proliferation followed by cartilaginous metaplasia [2]. The condition shows a clear male predominance and commonly affects individuals in their third to fifth decades of life [3].

Shoulder involvement is distinctly uncommon, particularly in young adults, and may present with chronic shoulder pain, stiffness, and reduced range of motion [4]. Early recognition is essential to prevent diagnostic delay and guide appropriate surgical intervention [5]. We report a rare case of primary synovial chondromatosis of the shoulder in a young adult managed successfully with arthroscopic excision, highlighting the diagnostic challenges and excellent functional outcome associated with minimally invasive management.

## Case presentation

A 25-year-old right-hand-dominant man presented with a long-standing history of right shoulder pain that had gradually progressed since his teenage years, without any antecedent history of trauma. He worked as a manual laborer, frequently engaged in heavy lifting and carrying, which he reported often exacerbated his symptoms. Multiple courses of conservative management, including analgesics and physiotherapy, failed to provide lasting relief. He denied any symptoms of instability, locking, or mechanical catching. The patient had no history of smoking, alcohol intake, recreational drug use, or relevant family history. He had no known comorbidities, including diabetes mellitus, inflammatory arthritis, or autoimmune disease.

On examination, there was no visible deformity or swelling. Active range of motion was reduced compared to the contralateral side, with flexion and abduction limited to 120°, external rotation to 30°, and internal rotation reaching the posterior superior iliac spine. All clinical tests for rotator cuff tear, shoulder instability, and impingement were negative. Distal neurovascular examination was intact. These findings were suggestive of a chronic intra-articular pathology without evidence of rotator cuff insufficiency or instability.

Investigations

**Figure 1:**
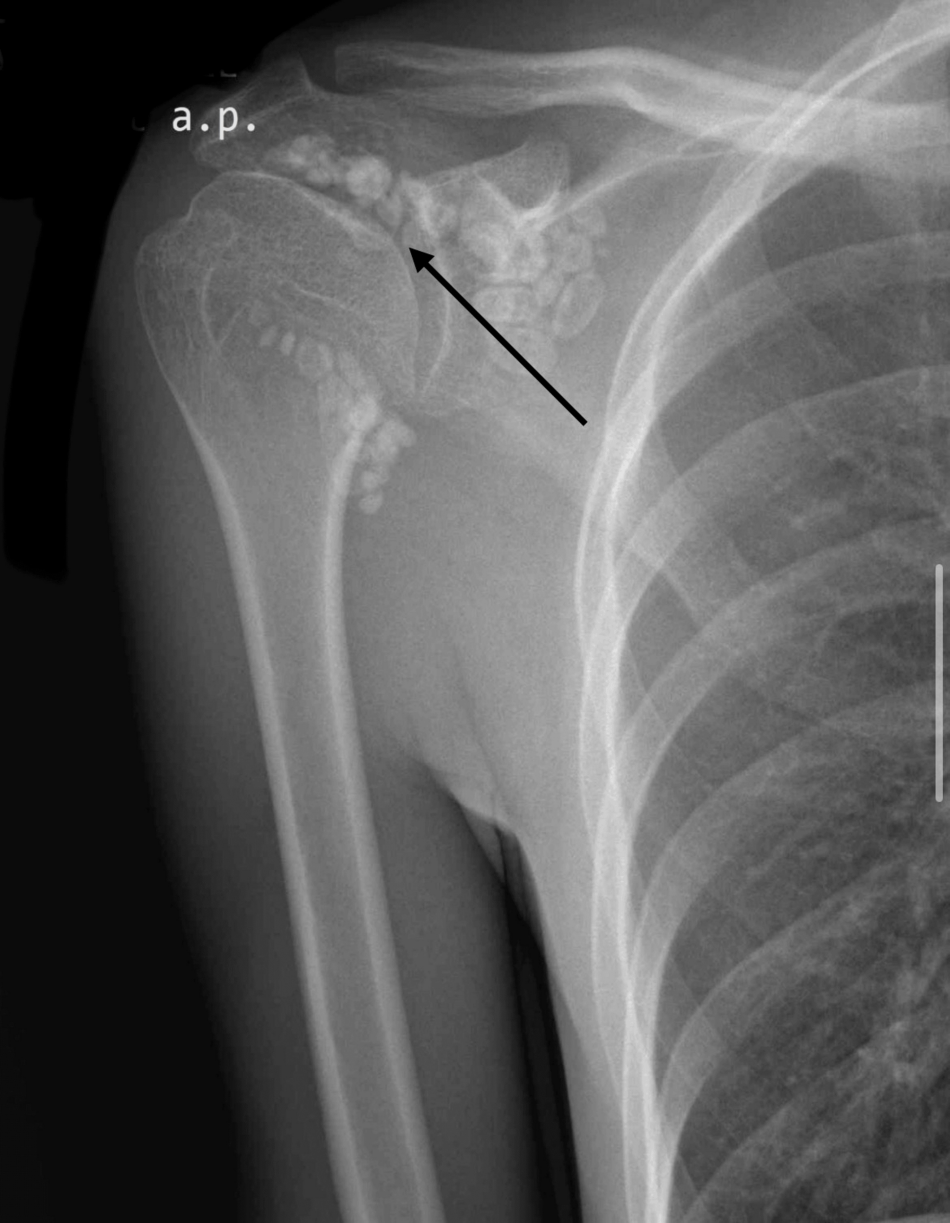
Preoperative Radiograph Preoperative anteroposterior radiograph of the shoulder showing intra-articular calcified bodies (Marked with arrow).

Plain radiographs of the shoulder revealed multiple well-circumscribed calcified bodies around the shoulder joint (Figure 1).

**Figure 2:**
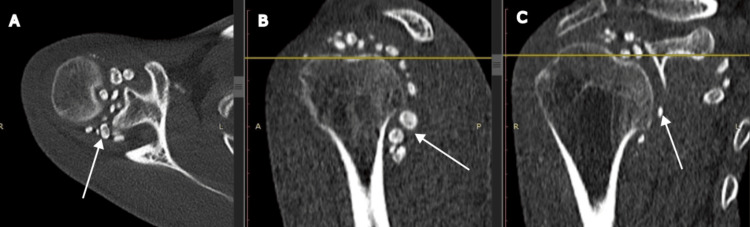
Non-Contrast CT (NCCT) Scan Multiple intra-articular calcified loose bodies in the shoulder joint in axial (A), sagittal (B), and coronal (C) views.

A non-contrast computed tomography (CT) scan of the shoulder demonstrated multiple intra-articular calcified loose bodies suggestive of synovial chondromatosis (Figure 2).

Treatment

**Figure 3:**
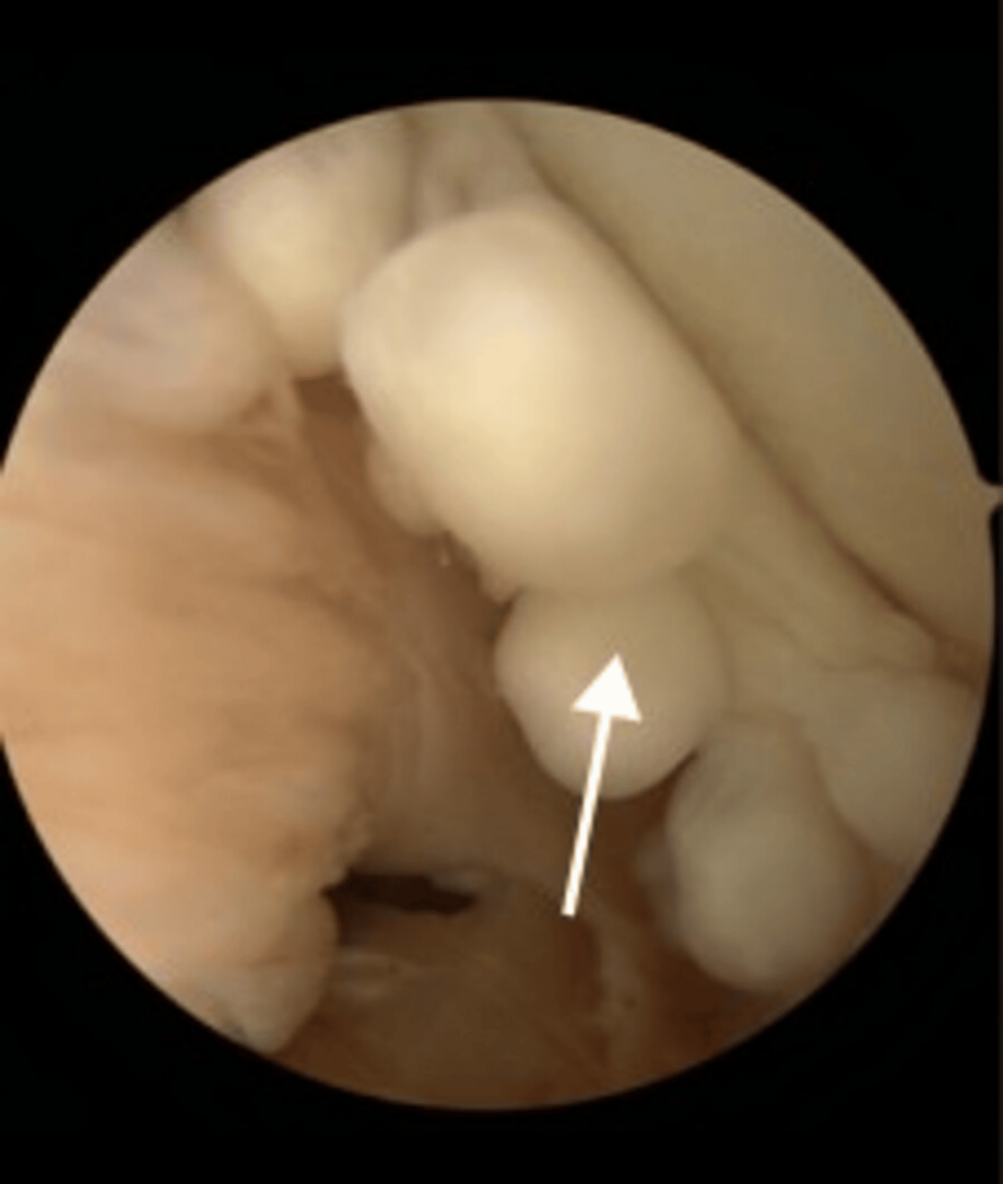
Intra-Operative Arthroscopic Image Arthroscopic images showing intra-articular nodules suggestive of synovial chondromatosis within the glenohumeral joint space.

**Figure 4:**
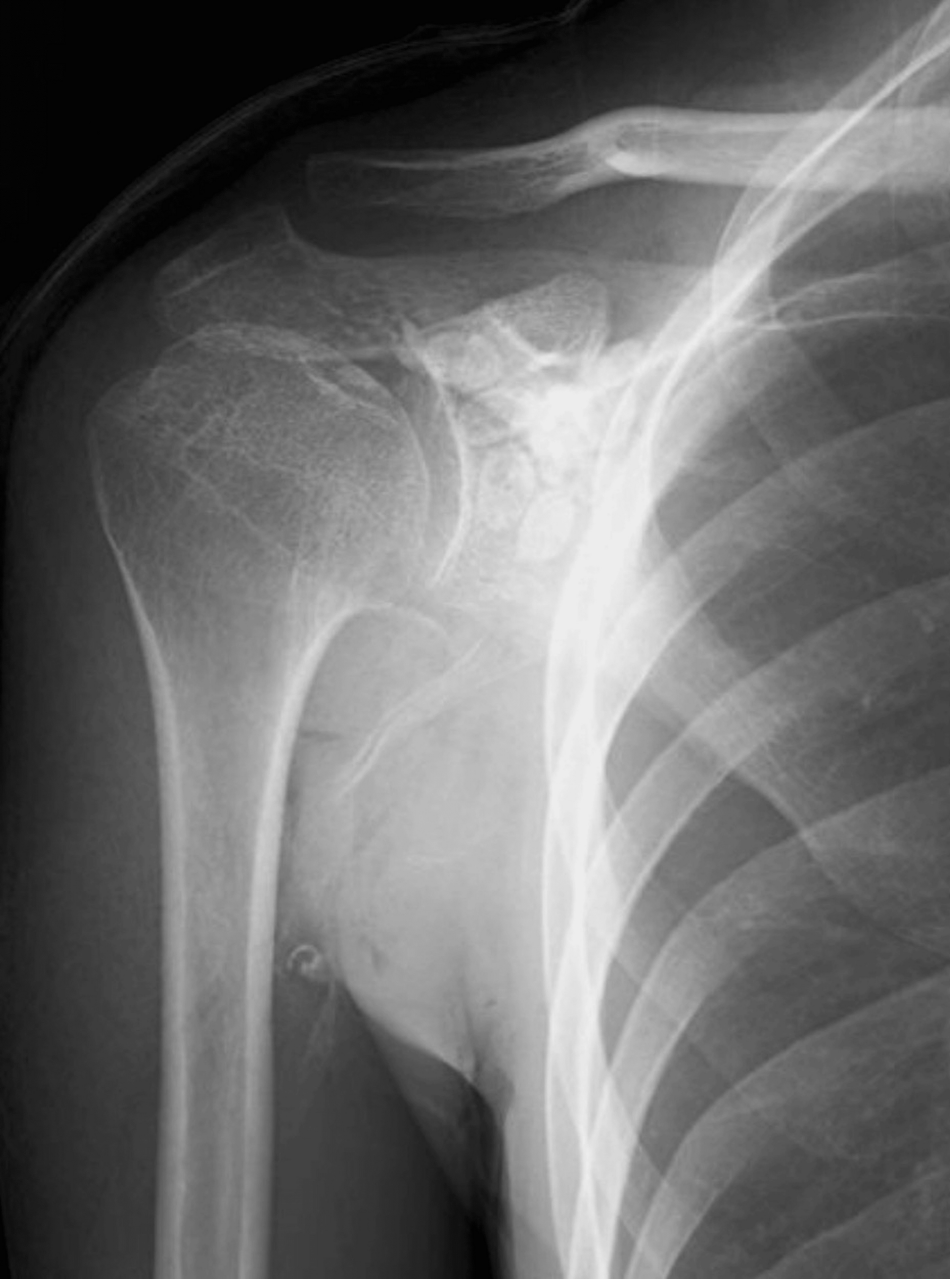
Postoperative Radiograph Postoperative radiograph showing complete removal of intra-articular calcified bodies.

The patient underwent diagnostic arthroscopy and arthroscopic removal of synovial chondromatosis with synovectomy. Under general anesthesia in the lateral decubitus position, a posterior portal was established to visualize the intra-articular fragments (Figure 3). Anterosuperior and anteroinferior portals were then used to remove the fragments with a grasper. Approximately 20 fragments were retrieved, and samples were sent for histopathological examination (Figure 4).

Histopathology revealed nodules of well-differentiated hyaline cartilage within the synovium, with areas of endochondral ossification and no evidence of atypia, confirming the diagnosis of synovial chondromatosis.

Outcome and follow-up

The patient had an uneventful recovery, with passive and active range of motion exercises started on the first postoperative day, followed by structured physiotherapy. At three months, he was pain-free with full shoulder motion and had returned to full occupational duties. No clinical or radiological recurrence was noted at the one-year follow-up.

## Discussion

Synovial chondromatosis is a rare benign disorder of the synovium, most commonly affecting the knee, followed by the hip and elbow, and only infrequently involving the shoulder. It predominantly affects adult males in the third to fifth decades of life and typically presents as a monoarticular process [1-4].

The condition often presents with non-specific symptoms such as chronic pain and limited range of motion, which can lead to delayed diagnosis, particularly in the early stages when cartilaginous nodules are not yet calcified and may be missed on plain radiographs. Advanced imaging modalities such as CT or MRI are useful in detecting non-calcified intra-articular nodules, while histopathological examination typically demonstrates well-differentiated hyaline cartilage nodules, occasionally undergoing endochondral ossification, with no cellular atypia [5,6].

Surgical removal of loose bodies with synovectomy remains the mainstay of treatment. Open procedures have historically been considered the gold standard, allowing complete removal from all recesses of the joint, including challenging areas such as the sub coracoid region or biceps sheath. However, open surgery is associated with increased morbidity, longer rehabilitation, and potential compromise of surrounding structures [4,7]. Arthroscopic management offers the advantages of less invasiveness, superior visualization, and faster recovery. Reported outcomes in shoulder cases are generally favorable, with successful results in the majority of patients, although recurrence occurs in a subset, particularly when nodules involve tendon sheaths [5,7]. Recurrence rates following surgery are estimated at up to 32%, most often related to incomplete synovectomy [8]. Malignant transformation to synovial chondrosarcoma is exceedingly rare, highlighting the importance of histological confirmation of excised tissue [9].

In the present case, the lesion was confined to the glenohumeral joint without involvement of the tendon sheath or bursa, allowing complete arthroscopic removal of approximately 20 loose bodies with partial synovectomy. This minimally invasive approach enabled rapid recovery, restoration of full shoulder function, and return to occupational activities without restriction. The case underscores the importance of considering synovial chondromatosis in the differential diagnosis of chronic shoulder pain, even in younger adults, and demonstrates the effectiveness of arthroscopic management for isolated intra-articular lesions.

## Conclusions

This case emphasizes the need to consider synovial chondromatosis in young adults presenting with chronic shoulder pain. Arthroscopic excision with synovectomy is a minimally invasive and effective treatment, allowing rapid recovery and restoration of function. Careful postoperative follow-up is essential, as incomplete synovectomy may increase the risk of recurrence.
